# Structural Features of Coumarin-1,2,4-Triazole Hybrids Important for Insecticidal Effects Against *Drosophila melanogaster* and *Orius laevigatus* (Fieber)

**DOI:** 10.3390/molecules30081662

**Published:** 2025-04-08

**Authors:** Domagoj Šubarić, Vesna Rastija, Maja Karnaš Babić, Dejan Agić, Ivana Majić

**Affiliations:** Faculty of Agrobiotechnical Sciences Osijek, Josip Juraj Strossmayer University of Osijek, Vladimira Preloga 1, HR-31000 Osijek, Croatia; dsubaric@fazos.hr (D.Š.); mkarnas@fazos.hr (M.K.B.); dagic@fazos.hr (D.A.); imajic@fazos.hr (I.M.)

**Keywords:** coumarin-1,2,4-triazole hybrids, insecticidal activity, *Drosophila melanogaster*, *Orius laevigatus*

## Abstract

Although the present use of pesticides in plant protection has limited the occurrence and development of plant diseases and pests, resistance to pesticides and their environmental and health hazards indicates an urgent need for new active ingredients in plant protection products. Recently synthesized coumarin-1,2,4-triazole hybrid compounds have been proven effective against plant pathogenic fungi and safe for soil-beneficial bacteria. *Drosophila melanogaster*, the common fruit fly, has been used as a model organism for scientific research. Additionally, it is considered a pest since it damages fruits and serves as a carrier for various plant diseases. On the contrary, *Orius laevigatus* is a beneficial true bug that biologically controls harmful arthropods in agricultural production. In the present study, we performed an adulticidal bioassay against *D. melanogaster* and *O. laevigatus* using coumarin-1,2,4-triazole hybrids. Quantitative structure–activity relationship studies (QSARs) and in silico ecotoxicity evaluation elucidated the structural features underlying the compounds’ insecticidal activity. The derivative of 4-methylcoumarin-1,2,4-triazole with a 3-bromophenyl group showed great insecticidal potential. A molecular docking study indicated that the most active compound probably binds to glutamate-gated chloride channels.

## 1. Introduction

Applying synthetic compounds in plant protection has numerous limitations due to their environmental and health hazard effects. The occurrence and development of plant diseases and pests, as well as resistance to pesticides, have led to the discovery of new active ingredients for plant protection products. The compounds should be highly specific, environmentally and toxicologically acceptable, and have the least impact on human health and non-target, beneficial organisms [1].

*Drosophila melanogaster* (Diptera: Drosophilidae), the common fruit fly, is used as a model to understand human biology and disease processes [2]. It is also a pest that causes a serious economic threat since it damages soft-skinned fruits by piercing the skin of matured fruits and laying eggs on them, promoting their microbial decay. The infected fruits could also carry various pathogenic microorganisms, such as *Staphylococcus aureus*, making them unsafe for consumption [3,4]. Sour rot is an economically important disease of grapes caused by an interaction of yeast and acetic acid bacteria, vectored by *Drosophila* spp. [5]. Additionally, the well-defined genetics of *D. melanogaster* allow it to be a model organism for screening pest insect targets, toxicity, and resistance of potential active substances of plant protection products [6,7,8,9,10].

*Orius laevigatus* (Fieber) (Hemiptera: Anthocoridae) is a zoo phytophagous predator widely used in pest management strategies. This beneficial insect also preys on other key agricultural pests, such as thrips, whiteflies, aphids, and spider mites. In addition, it repels pests and natural enemies, contributing to plant-induced defenses [11,12].

Coumarin-1,2,4-triazole hybrids were previously synthesized by the green approach using choline chloride: urea deep eutectic solvent [13]. Recent studies have shown that these compounds inhibit the growth of plant pathogenic fungi *Sclerotinia sclerotiorum* and *Fusarium oxysporum* but not of soil-beneficial bacteria, making them promising candidates for further research in plant protection [14]. 1,2,4-Triazoles have proven insecticidal activity, as derivatives containing trifluoroacetyl moieties have shown activity against brown planthopper (*Nilaparvata lugens*) and cowpea aphid (*Aphis craccivora*) [15]. Meta-diamide compounds containing 1,2,4-triazole showed good insecticidal activity against the diamondback moth (*Plutella xylostella*) and oriental armyworm (*Mythimna separate*) [16].

Prompted by the above results, this study aimed to evaluate the insecticidal effect of the coumarin-1,2,4-triazole hybrids on the common fruit fly and beneficial organism *O. laevigatus*. The structural features important for the insecticidal activity were investigated by a quantitative structure–activity relationship (QSAR) study.

In the ecotoxicological modeling and design of agrochemicals, computer-aided molecular design has become widely accepted and used. REACH (Registration, Evaluation, and Authorization of Chemicals) guidelines published by the European Chemicals Agency (ECHA) in 2011 [17] state that animal tests can be avoided if the hazardous properties of a substance can be predicted using quantitative structure–activity relationships (QSARs). Toxicological and environmental property evaluation using computational approaches saves time and the economic costs of synthesis and biological tests. The VEGA application, like numerous open-source tools and standalone applications, uses QSAR models for the prediction of properties, categorized in several groups, such as human toxicity, eco-toxicity (bioconcentration factor; aquatic acute and chronic toxicity; terrestrial acute toxicity; sludge toxicity, zebra fish embryo activity), and fate and distribution in the environment [18,19].

In our previous study, coumarin-1,2,4-triazole hybrids showed weak to moderate inhibitory potential against acetylcholinesterase (AChE) [13]. Potent insecticide spinosad, a naturally occurring mixture of two active components, spinosyns A and D, shows great selectivity toward target insect orders Coleoptera, Diptera, Homoptera, Hymenoptera, Isoptera, etc. Also, it shows weak activity against many beneficial organisms and reduced risk toward mammals and many other aquatic and avian animals [20,21,22]. The exact mechanism of action of spinosyns is still not known with certainty, but it is well known that it causes widespread excitation of neurons in the central nervous system [23]. Some studies suggest that they have potent effects on nicotinic acetylcholine receptors (nAChRs), leading to disruption of acetylcholine neurotransmission, the insect γ-aminobutyric acid (GABA) receptor function, or invertebrate glutamate-gated chloride channels (GluCls) [24,25,26,27]. A molecular docking study will elucidate the possible mode of action of cucoumarin-1,2,4-triazole hybrids.

## 2. Results

### 2.1. Structure of Compounds

**Scheme 1:**
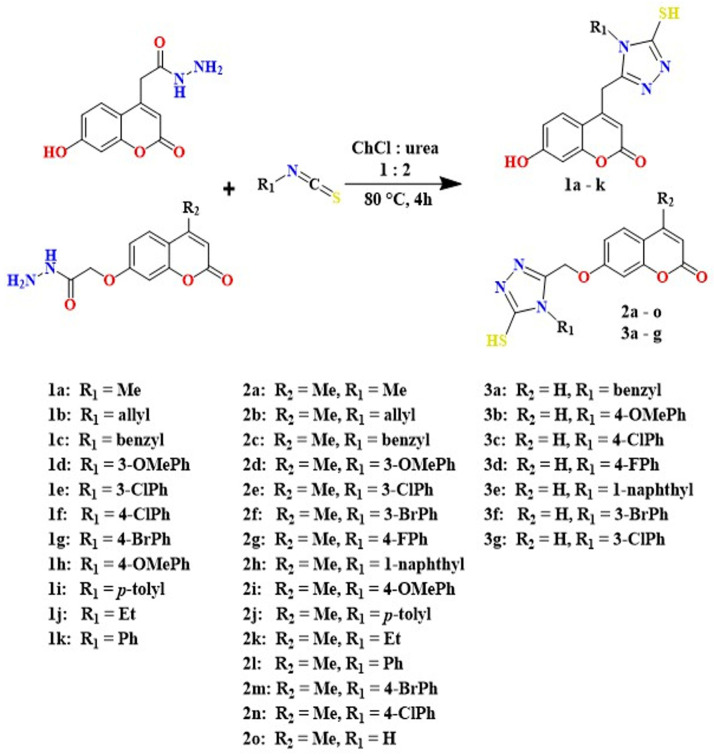
One-step “green” synthesis of coumarin-1,2,4-triazoles in deep eutectic solvent and structures of coumarin-1,2,4-triazole hybrids.

Synthesis of analyzed coumarin-1,2,4-triazole hybrids was as described previously [13,28]. Briefly, the compounds were synthesized in a one-step reaction by a green approach using choline chloride: urea deep eutectic solvent. Scheme 1 presents the synthesis procedure and the structure of the compounds.

### 2.2. Insecticidal Activity

The results of the mortality bioassay for *D. melanogaster* and *O. laevigatus* are presented in Table 1. The effect of coumarin-1,2,4-triazole hybrids on the mortality of *D. melanogaster* was observed over eight days and compared with the positive control (insecticide spinosad). After two days, neither compound showed statistically significant mortality similar to spinosad, but the highest effect was shown by compound **2f** (60.30%). Four days after treatment, only the same compound, **2f**, exhibited mortality statistically similar to insecticide (86.36%). After eight days, except compound **2f,** which caused the highest mortality of *D. melanogaster* (100%), compounds **1b**, **1c**, **1g**, **1i**, **1k**, **2b,** and **2n** also exhibited significantly similar mortality as spinosad. These compounds were evaluated for the toxicity bioassay on the beneficial predator insect *O. laevigatus* (Fieber) (Table 1). Among these compounds, only **1g**, **1h**, and **2f** caused significantly lower mortality than spinosad. For instance, the lowest mortality against *O. laevigatus* (Fieber) after 48 h was shown in compound **1g** (64.45%) and **2f** (61.67%), while the spinosad killed 100% of the animals.

### 2.3. Predicted Environmental Fate Properties and Ecotoxic Effects

Table 2 shows the environmental fate properties and ecotoxic effects calculated by the QSAR-based model using the VEGA application. The Registration, Evaluation, Authorisation and Restriction of Chemicals (REACH) regulation [17] identified a substance as bioaccumulative if log BCF > 3.301 and very bioaccumulative when log BCF > 3.699. The studied compounds have a low bioaccumulation potential, but for the 4-fluorophenyl derivative, **2g**, the highest value (BCF = 1.35). The highest acute toxicity to fish was predicted for the compounds **2h** and **3e** (pLC_50_ = 4.8 mmol/L), which possess 1-naphthyl substituent at the R_1_ position of the triazole ring (Scheme 1). Compound **2h** also had the highest potential for killing fathead minnows 96 h after treatment (pLC_50_ = 6.49 mol/L). The highest developmental toxicity toward zebrafish embryos was predicted for compounds **3a**–**3c** (half-maximal activity concentration, AC_50_, 0.41–0.44 µmol/L). The strongest short-term toxicity (48 h) to planktonic freshwater crustacean *Daphnia magna* was predicted for two derivatives with 3-bromophenyl substituent at the R_1_ position of the triazole ring (**3f**, pLC_50_ = 7.02 mol/L; **2f** = 6.99). Only five compounds were classified as non-toxic to freshwater algae and cyanobacteria (**1b**, **1d**, **1h**, **1k**, and **3b**). Most compounds were predicted as non-toxic for the honeybee, except for **3g**. Toxic effects on microorganisms from activated sludge were predicted for compounds **1e**, **1f**, **1i**, **1k**, and **3d**–**3k**.

### 2.4. QSAR Study of Mortality of D. melanogaster

Multiple linear regression (MLR) QSAR models were built for the mortality of *D. melanogaster* (MDM) observed four days after treatment. It was not possible to find a linear predictive three-parametric model that satisfied both internal and external validation criteria. However, the best obtained MLR model is as follows:

log MDM = 19.72 − 32.11 (−0.47) *PW2* + 0.03 (0.29) *RDF075m* + 0.49 (0.66) *nRCt(sp2)* (1)

*N* (training) = 33; *R*^2^_tr_ = 0.51; *R*^2^_adj_ = 0.46; *Q*^2^_LOO_ = 0.36; *Kxx* = 0.21; Δ*K* = 0.06; *F* = 9.94; *RMSE_tr_* = 0.21; *RMSE_cv_* = 0.24; *Q*^2^_LOO_ = 0.36 *CCC_tr_* = 0.67; *CCC_cv_* = 0.57; *MAE_tr_* = 0.17; *MAE_cv_* = 0.20; *R*^2^_scr_ = 0.09; *Q*^2^_scr_ = −0.18

The relative importance of descriptors in the model is assessed by beta coefficients (in brackets). The obtained model contains three molecular descriptors that are not mutually correlated according to values of global correlation among descriptors (Δ*K* > 0.05) and global correlation among descriptors (*Kxx* < 0.4). However, the QSAR model failed in fitting criteria since the coefficient of determination of the training set (*R*^2^_tr_), adjusted (*R*^2^_adj_), and cross-validated *R*^2^ using the leave-one-out method (*Q*^2^_LOO_) are too low. Also, concordance correlation coefficients of the training set (*CCC_tr_*) and test set using LOO cross-validation (*CCC_cv_*) are lower than 0.80. However, the values of root-mean-square error of the training set (*RMSE_tr_*) and root-mean-square error of the training set determined through the cross-validated LOO method (*RMSE_cv_*) have approximately equal values, as well as mean absolute error of the training set (*MAE_tr_*), internal validation set (*MAE_cv_*), and external validation set (*MAE_ex_*). The values of both coefficients, *R*^2^_scr_ (Y-scramble correlation coefficients) and *Q*^2^_scr_ (Y-scramble cross-validation coefficients) were <0.02, implying that the models were not obtained by chance [29,30].

Since the generation of an MLR model that satisfied the criteria of the internal and external validation failed, we may conclude that a linear relationship between MDM and the structure of coumarin-1,2,4-triazole hybrids does not exist. To find a nonlinear relationship between the mortality of *D. melanogaster* and the structures of coumarin-1,2,4-triazole hybrids derivatives and improve the model’s predictability, we performed an artificial neural network (ANN) approach [31]. The statistical parameters of the best multilayer perceptron (MLP) ANN model obtained are shown in Table 3.

**Figure 1:**
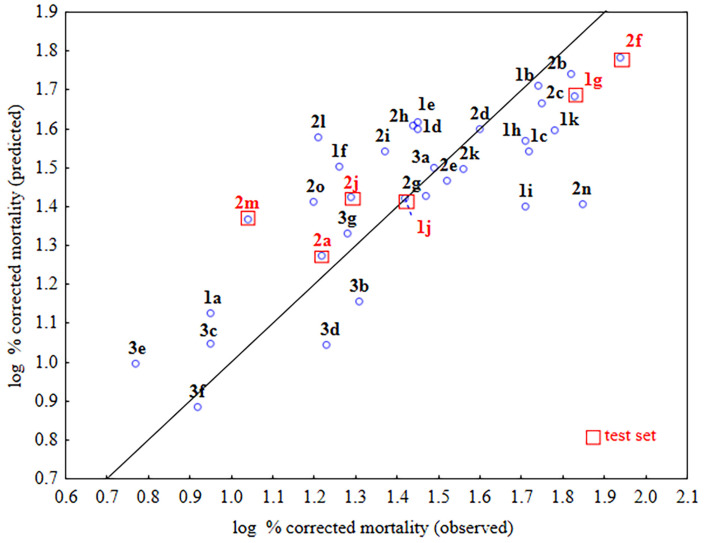
The plot of the experimental values of log % mortality of *D. melanogaster* (MDM) observed four days after treatment, vs. calculated by an artificial neural network model MLP 3-4-1.

The importance of each variable in the MLP-ANN model 3-4-1 is revealed through global sensitivity analysis (Table 4) [32]. Since the global sensitivity should be large (>>1) if the variable is important, we may conclude that the most important variable in the model is the number of aliphatic tertiary C atoms attached to the sp^2^ C atom (*nRCt(sp2)*). Path/walk 2-Randic shape index (*PW2*) is the second most important descriptor. Regarding order of importance, each descriptor in the MLP-ANN 3-4-1 model corresponds to the beta coefficients in MLR model (1). The correlation between the experimental and calculated values of log MDM for the training and test set is shown in Figure 1 (see figure below).

### 2.5. Interactions with Targets Related to Insecticidal Activities

To elucidate the possible mechanism of coumarin-1,2,4-triazole hybrids for the insecticidal activity of *D. melanogaster,* a docking study was performed on glutamate-gated chloride (CGluCl) channels as a target protein (pdb: 3RIF). The compounds docked into the binding site of the antiparasitic drug ivermectin, which was co-crystallized with the GluClcryst–Fab complex. Ivermectin (**IVM**) acts as an allosteric agonist activating invertebrate glutamate-gated chloride channels [26]. Molecular docking was also performed on *D. melanogaster* dopamine transporter (dDAT), which is structurally related to GABA transporter isoforms (GATs) (pdb: 7WGT) [33] and chimeric acetylcholine binding protein from *Aplysia californica* (Ac-AChBP) (pdb: 4ZK4) [34].

The five best docking score energies (kcal/mol) of coumarin-1,2,4-triazole hybrids on the GluClcryst–Fab complex, (pdb: 3RIF), *D. melanogaster* dopamine transporter (dDAT) (pdb: 7WGT), and acetylcholine binding protein from *Aplysia californica* (Ac-AChBP) (pdb: 4ZK4), along with the co-crystallized ligands (ivermectin, **IVM**; **NO-711**; **TII**), and an active component of pesticide (spinosyn A, **SPYNA**), are tabulated in Table 5.

**Figure 2:**
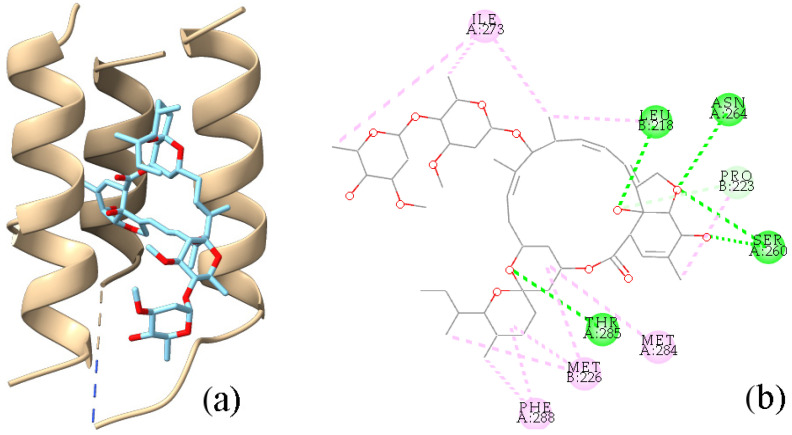
(**a**) Docked pose of ivermectin (IVM) in glutamate-gated chloride channels (pdb: 3RIF). (**b**) Two-dimensional representation of the main interactions. (Green: hydrogen bond; light green: carbon–hydrogen bond; light pink: alkyl and *π*-alkyl interactions).

**Figure 3:**
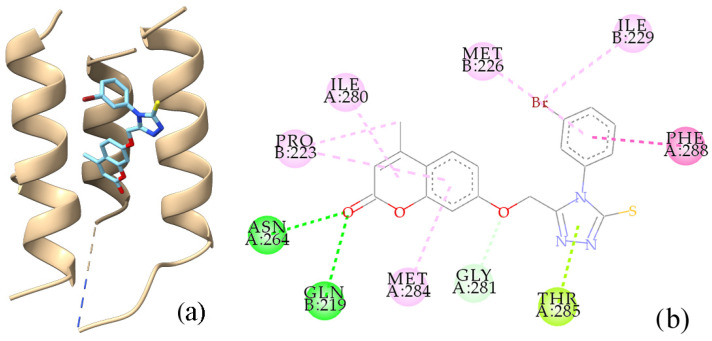
(**a**) Ivermectin-binding site in glutamate-gated chloride channels docked with compound **2f** (pdb: 3RIF). (**b**) Two-dimensional representation of the main interactions. (Green: hydrogen bond; light green: π-lone pair; very light green: carbon–hydrogen bond; pink: π-π T-shaped; light pink: alkyl and *π*-alkyl interactions.)

The five compounds with the highest mortality against *D. melanogaster* (**1c, 1g**, **2b, 2f, 2n**) showed affinities for binding to different targets. Compound **2f** realized the highest energy binding in the IVM-binding site located at the transmembrane domain of the receptor inside of four α-helical transmembrane spans (M1–M4) (Figure 2a and Figure 3a). Two-dimensional diagrams of the interactions of ligands with amino acid residues with IVM and **2f** are presented in Figure 2b and Figure 3b. Compound **2f** shares a similar interaction pattern as **IVM,** making a hydrogen bond with Thr285 and Asn264, both through oxygen atoms from the coumarin-carbonyl group. Through interaction with Thr285, which **IVM** makes by hydrogen bond via oxygen atom from the spiroketal motif, **2f** is generated by a π-lone pair interaction from the triazole ring. Compound **2f** also has a very important structural feature, the methyl group at position R_2_ of the coumarin core. This leads to van der Waals interactions with Pro222 and bromine atoms with Met226 and Ile229 [26].

**Figure 4:**
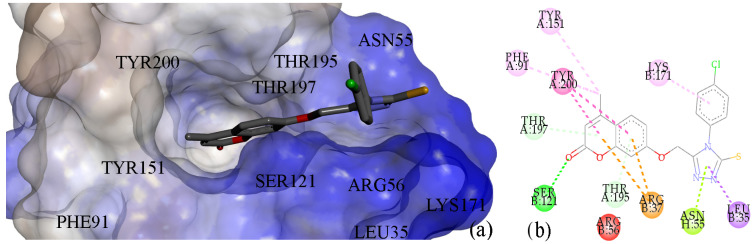
(**a**) Hydrophobic surface representation (from 3.0 as brown to −3.0 as blue) of the glutamate-binding site of glutamate-gated chloride channels (pdb: 3RIF) with docked compound **2n**. (**b**) Two-dimensional representation of compound **2n**’s interactions with residues (green: hydrogen bond; light green: π-lone pair; very light green: π-donor hydrogen bond; purple: π-π T-shaped; pink: π-π stacked; light pink: *π*-alkyl; orange: π-cation; red: unfavorable interactions).

Compounds **2n** and **2b** also show a high tendency for binding to GluCl, but in the glutamate binding site located at the N-terminal extracellular domain, lodged between subunits, consisting of mostly of β-structure. Figure 4a presents the docking pose of compound **2n** into the glutamate binding site. The binding site is located in loop C. Its sides are built with loops from the (+) subunit with Tyr15 and Tyr200, and its base is from β strands on the (−), containing Arg37 and Arg56 [26]. The main interactions with the amino acid residues of the binding site with the compound **2n** are presented in a two-dimensional diagram (Figure 4b). Similar structural features to compound **2f** allow compound **2n** binding: the oxygen atom from the coumarin-carbonyl group for the creation of a hydrogen bond with Ser121, the methyl group at the position R_2_ of the coumarin core, and the free π-electron from the triazole ring for the formation of van der Waals interactions. Spinosyn A shows a very weak binding affinity to the GluClcryst–Fab complex, so we may conclude that it does not act as an allosteric ligand for glutamate-gated chloride channels.

**Figure 5:**
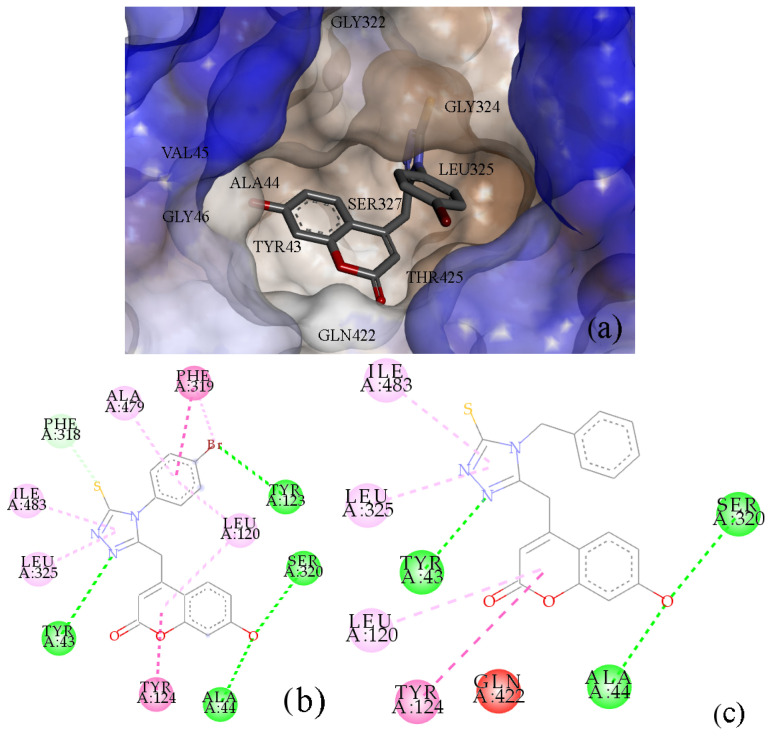
(**a**) Docked pose of the compound **1g** into the binding pocket of *D. melanogaster* dopamine transporter (pdb: 7WGT) represented by a hydrophobic surface (from 3.0 as brown to −3.0 as blue); (**b**,**c**) two-dimensional diagram of the major interaction of compounds **1g** and **1c**, respectively. (Green: hydrogen bond; very light green: π-donor hydrogen bond; pink: π-π T-shaped; light pink: π-alkyl; red: unfavorable interactions.)

Compounds **1g** and **1c** show affinities to bind to the *D. melanogaster* dopamine transporter, indicating their possible role as inhibitors of GABA transport, the major neurotransmitter in the synaptic space [33]. Figure 5a shows the binding pocket of *D. melanogaster* dopamine transporter, the binding site of the inhibitor NO-711 (pdb: 7WGT) represented by a hydrophobic surface. The major interactions with residues of the binding site with compounds **1g** and **1c** are shown in Figure 5b and Figure 5c, respectively. Although both compounds create hydrogen bonds with Ser320 and Ala44 through the hydroxyl group of the coumarin core, as with Tyr49 via nitrogen from the triazole ring, the compound can create one more hydrogen bond between 4-Br from the phenyl ring and Tyr49.

The bromine atom also creates π-π T-shaped and π-alkyl interactions with Phe319 that additionally further strengthen the binding into the binding pocket of the *Drosophila melanogaster* dopamine transporter.

Compounds with the highest mortality against *D. melanogaster* did not show the potential for binding to the acetylcholine binding protein from *Aplysia californica* (Ac-AChBP) (pdb: 4ZK4). Among these five compounds, **2b** binds with the highest energy (−97.62 kcal/mol), which is significantly weaker than the co-crystalized ligand TII (−116.98 kcal/mol) [34]. A very low binding potential was also found for Spinosyn A (−50.70 kcal/mol) (Table 5).

## 3. Discussion

Reliable assays are required to obtain data on the bioactivity of newly synthesized chemical compounds [35]. This is important for the development and risk assessment of novel pest control tactics. This study evaluated the mortality effects of various newly synthesized chemical compounds on two distinct insect species, *D. melanogaster* and *O. laevigatus*, using different methods of exposure. Both of our bioassays tested the toxicity of the test compound provided in the artificial diet for *D. melanogaster* and contact toxic effects on *O. laevigatus*. The results demonstrate that the assays were able to detect the activity of the tested compounds on the survival of both tested insects. The compounds exhibited a range of efficacies, reflected in mortality rates that varied significantly across the species and the exposure times.

For *D. melanogaster*, the oral toxicity tests revealed that eight compounds, **1b, 1c**, **1g**, **1i**, **1k**, **2b**, **2f**, and **2n**, induced moderate to high mortality rates, approaching or reaching 100% over longer exposure periods. This suggests a potent efficacy of these compounds when ingested by the flies, likely affecting vital physiological or metabolic pathways. Interestingly, the pattern of mortality across days indicates a cumulative toxic effect, with significantly higher mortality recorded at each successive time point.

In contrast, the contact toxicity tests with *O. laevigatus* showed a different trend. Compounds such as **1b**, **1c**, and **2n** again demonstrated high mortality, but the speed of action was remarkable, achieving total mortality within 72 h. This rapid effectiveness might be due to the compounds’ properties, which facilitate quick absorption and action upon direct contact, possibly interfering with the insects’ cuticular structure or neural functions.

The variability in response between the two species under different exposure scenarios is noteworthy. However, the outcomes of the bioassays were comparable. The same compounds had similar effects on both the pest and the beneficial insect. While some compounds (e.g., **1b** and **1c**) showed moderate to high mortality rates for both species, others (e.g., **2f**) were less effective, suggesting species-specific resistance or differing mechanisms of action. As a result of differential sensitivity, targeted pest management strategies that consider pest species’ biological and behavioral characteristics are necessary.

Moreover, the safety profile of these compounds must be considered. The high efficacy observed with compounds like **1b** and **1c** against both tested insect species could be advantageous for suppressing a broad spectrum of insects. However, potential non-target effects, especially on beneficial insects like *O. laevigatus*, which are natural enemies of pests, necessitate a cautious approach. The designed compounds minimize impacts on non-target species while maximizing pest control efficacy. For sustainable and integrated pest management (IPM) approaches, the aim is not always to achieve 100% mortality but rather to reduce pest populations to below damaging levels while minimizing the impact on non-target species and the environment. Therefore, determining the sublethal and lethal impacts of these compounds provides critical insights into their suitability for pest management.

For compounds where *O. laevigatus* was tested, it is critical to discern which ones might be promising for selective pest management due to their minimal impact on non-target organisms. Compounds **1g** and **2f** showed relatively lower mortality rates at earlier time points (24 h) before reaching higher mortality by 72 h. This suggests that these compounds may initially induce sublethal effects, which could include physiological stress or reduced predatory efficiency, before culminating in death. We considered compounds sublethal at mortality rates less than 50%. At these levels, the effects may include changes in feeding behavior, reproduction rates, growth inhibition, or physiological stress which do not necessarily lead to immediate death but can impact population dynamics over time. Often considered are mortality rates that exceed 50%, with higher thresholds like 70% or 90% frequently used in studies to denote effective lethal concentrations. For regulatory purposes or practical pest control, a 90% mortality rate is often used as a benchmark for considering a compound highly effective.

Compounds **1b**, **1c**, and **2n** exhibited high mortality rates in *O. laevigatus*, achieving near 100% mortality by 72 h. These results are indicative of a strong lethal effect, which, while effective for pest control, might pose risks to beneficial insects if used without careful management strategies that mitigate exposure to non-target species.

For *D. melanogaster*, the range of responses also varied significantly. Compound **2f** showed very high mortality rates 2 days after exposure, consistently increasing over time and culminating at 100% by 8 days. These are clear indicators of lethal effects. Compounds such as **2c** demonstrated moderate mortality rates which did not reach as high as 8 days, suggesting a possible sublethal impact at lower or earlier exposure levels.

Given our aim to identify compounds with minimal impact on non-target organisms, it is crucial to focus future testing on compounds that show promise in selectively affecting target pests without harming beneficial species like *O. laevigatus*.

According to the obtained results, compound **2f** stood out as the leading compound due to very high mortality rates 2 days after exposure to *D. melanogaster*, and the lowest lethal impact on a beneficial insect, *O. laevigatus*. Moreover, this compound has been predicted to be low in bioaccumulative activity, low acute toxicity to fish, mediumly toxic to zebrafish embryos, and non-toxic to honeybees and microorganisms from activated sludge. However, predictive models warned that compound **2f** has possible short-term toxicity on planktonic organisms, such as *Daphnia magna*.

To elucidate the important structural feature important for the insecticidal effects of coumarin-1,2,4-triazole hybrids, QSAR analysis was performed. Very often, different QSAR problems, especially complex biological activities, such as toxicity on animals, involve some degree of non-linearity. That required choosing a non-linear QSAR method, such as neural networks [36]. The MLR QSAR model did not meet the validation criteria, but it selected the best input variables for ANN. The variable with the highest influence on the mode, according to global sensitivity analysis (Table 3), is a descriptor that belongs to the functional group accounts, the number of aliphatic tertiary C atoms attached to the sp^2^ C atom (*nRCt(sp*2*)*) [37]. Since compounds **3a**-**3g** lack this functional group, this descriptor strongly discriminates groups of compounds **1a**-**1k** and **2a**-**2o**. For comparison, compound **2f**, which differs in structure from compound **3f** only by possessing a methyl group attached to the phenyl ring at the R_1_ position (Scheme 1) caused 86.36% mortality of *D. melanogaster* after four days, while compound **3f**, which has only hydrogen at this position, killed only 8.37% (Table 1). Similarly, a difference could be observed between compound **2n** (71.44%) and **3c** (8.92%). Based on docking studies, it has been shown that methyl groups of compounds **2f** and **2n** are important for creating van der Waals interactions with glutamate-gated chloride channels (Figure 3b and Figure 4b). Interestingly, descriptor *nRCt(sp*2) was also involved in the QSAR model performed on the same set of compounds for the antifungal activity against *Sclerotinia sclerotiorum.* Analysis of the structure–activity relationship and the obtained QSAR model implied that aliphatic tertiary C atoms attached to the sp^2^ C atom enhance antifungal activity [14]. Benzo-4-methyl coumarin also showed a stronger effect against fungi *Alternaria solani* and *Fusarium oxysporum*, and bacteria *Erwinia amylovora* and *Ralstonia solanacearum* than 3-acetyl coumarin [38]. 4-Methyl-7-hydroxy coumarins are promising candidates as a biopesticide since they have shown insecticidal activity against larvae of the yellow fever mosquito (*Aedes aegypti*) and southern house mosquito (*Culex quinquefasciatus*) [39]. Through the inductive-stabilization effect, the methyl group donates electrons to the sp^2^ C atom of the coumarin benzene ring, enabling the creation of alkyl-alkyl and *π*-alkyl interactions with amino acid residues of the receptor. The second variable in the QSAR model is the topological descriptor, path/walk 2-Randic shape index (*PW2*), which represents the ratio between the atomic path and atomic walk topological length two. The descriptor is sensitive to the type of substituent on the coumarin or triazole core. A lower value of the *PW2* descriptor is found in compounds with substituents that negatively affect insecticidal activity. For example, compound **2f** with 3-BrPh in the 4-N position of the triazole ring has a *PW2* value of 0.59 (86.36% mortality 4. day), while compound **2h** with 1-naphthyl in the position has a *PW2* value of 0.589 (17.55% mortality 4. day). Similarly, compound **3f**, which possesses 3-BrPh at the 4-C position of the coumarin core, has a higher value of PW2 (0.587) and better mortality (8.37%) than compound **3e**, which in the same position has 1-naphthyl, a lower value of *PW2* (0.585) and lower insecticidal activity. Also, a similar effect can be observed in compounds with allyl and benzyl substituents that reduce insecticidal activity (Table 1). The third variable, *RDF075m*, which belongs to the Radial Distribution Function (RDF) group of descriptors, presents information on the three-dimensional distribution of mass in molecules within a radius of 7.5 Å from the geometric center of the molecule. The compound **2f** has the highest value of *RDF075m* (12.948), as well as the highest value of insecticidal activity. This high value of *RDF075m* of compound **2f** could be attributed to the Br atom that fits inside a radius of 7.5 Å.

The presence of a Br atom in compounds has shown varying effects depending on the target pest and molecular structure. Brominated derivatives exhibit enhanced insecticidal properties compared to their non-brominated counterparts. Specifically, certain Br compounds demonstrated higher mortality rates against *Mythimna separata* larvae, suggesting that introducing a Br atom at the furan site of fraxinellone significantly boosts its insecticidal efficacy [40]. Another example, the meta-diamide insecticide broflanilide, which contains a Br atom, exhibited high efficacy against Lepidopteran pests and pests from other insect orders by modulating GABA (gamma-aminobutyric acid) receptors [41]. The IRAC classified broflanilide as a member of a new group, Group 30, and its mode of action is a GABA-gated chloride channel allosteric modulator. In insects, the GABA-gated chloride channel plays a crucial role in the nervous system by regulating nerve signal transmission. When GABA binds to this receptor, it opens chloride channels, allowing chloride ions to flow into nerve cells, which reduces their activity and prevents overstimulation. Allosteric modulators, like certain insecticides, do not bind directly to the GABA site but instead attach to a different part of the receptor, enhancing or disrupting its normal function. This can lead to excessive nerve inhibition, paralysis, and ultimately, insect death [42].

The molecular docking study provided insight into the possible mode of action of the insecticidal effect of the studied compounds. Compound **2f** shows a similar binding mode to ivermectin, an antiparasitic agent that binds to a GluCl channel common to invertebrate nerves and muscle cells. Binding to the receptor, ivermectin causes conformational changes that activate the opening channel for the flow of chloride ions, hyperpolarize the cell membranes, and kill the invertebrate. Very potent compound **2n** can also bind the GluCl channel, but at the glutamate binding site. It is considered that binding at this site stabilizes the open state of the receptor, increasing the chloride ion conductance [26,43]. For both compounds **2f** and **2n**, very important common moieties for interacting with receptor residues are methyl and carbonyl at the coumarin core, as 1-N and 2-N atoms from the triazole ring.

The neurotransmitter dopamine affects emotions, behavior, movement, and memory. The dopamine transporter (*DAT*) regulates dopamine signaling in the synaptic cleft [44]. *Drosophila* dopamine transporter (*dDAT*) belongs to the neurotransmitter-sodium symporter family, which includes transporters for serotonin, norepinephrine, and GABA that utilize the Na^+^ gradient to drive the uptake of substrate [45]. The highest affinity towards this receptor has two hybrids 7-hydroxycoumarin and 1,2,4-triazole (**1g**, **1c**), where exactly the 7-hydroxyl group is important for the generation of a strong hydrogen bond with Ala44 and Ser320.

**Figure 6:**
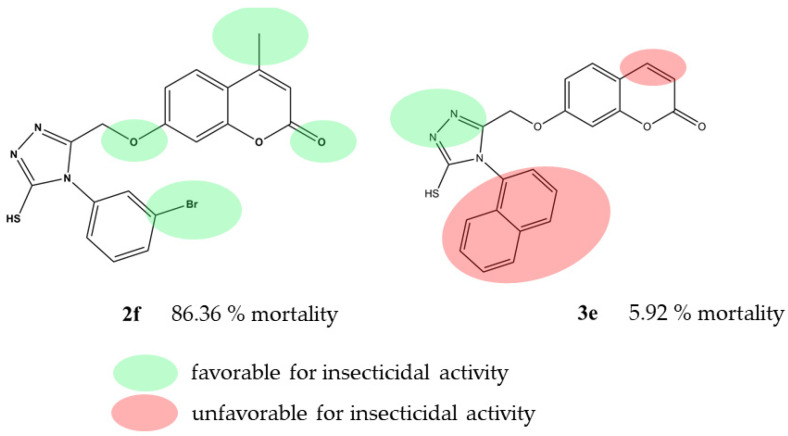
Structural features of coumarin-1,2,4-triazole hybrids favorable and unfavorable for the mortality of *D. melanogaster*.

Figure 6 illustrates the overall structural features of coumarin-1,2,4-triazole hybrids that affect their insecticidal activity compounds **2f** and **3e**, based on the results of the QSAR and molecular docking study.

## 4. Materials and Methods

### 4.1. Synthesis of Coumarin-1,2,4-Triazoles

The characterization and synthesis procedure of coumarin-1,2,4-triazoles as a one-step reaction was described previously. ^1^H NMR, ^13^C NMR, and mS spectra are available in the Supplementary Materials on the publisher’s website along with the published article (https://www.eurekaselect.com/article/133759, accessed on 3 April 2025) [13].

In brief, a mixture of the corresponding coumarin hydrazide (1.0 eq, 1.6 mmol) and various alkyl and aryl substituted isothiocyanates (1.25 eq, 2.0 mmol) was added to a deep eutectic solvent choline chloride:urea (1:2 molar ratio). The mixture was stirred at 80 °C, and the progress of the reaction was monitored using thin-layer chromatography (TLC). Once the reaction was complete, the mixture was allowed to cool to room temperature, after which water was added. The crude product was collected through filtration and then recrystallized in ethanol.

### 4.2. Bioassays

The bioassays were conducted under laboratory conditions and maintained at 23 ± 2 °C and a relative humidity of 65 ± 5% with a 12:12 h light/dark cycle.

#### 4.2.1. Toxicity Assay to *D. melanogaster*

Three-day-old adult flies were starved for 24 h before testing and placed in entomological cages. The oral toxicity assay was conducted in triplicate for each of the compounds, with 20 flies per repetition. The negative control feed was a standard water-sucrose-yeast mixture in a 50 mL:40 g:10 g ratio. The pesticide that was used as a positive control was a commercial formulation of spinosad (Laser^TM^, Corteva Agriscience^TM^, Zagreb, Croatia). Tested compounds were dissolved in dimethyl sulfoxide (DMSO) 10% *v*/*v*, and then diluted with water. The feed was prepared by mixing the standard water–sucrose–yeast feed with a pesticide or compound solution. Spinosad and tested compounds were prepared at 100 mg/L in the final feed mixture. The feed was administered into the cages on parafilm, where twenty 15 µL droplets were previously put using a micropipette. Flies were offered the opportunity to feed continuously for five days, after which the treatment feeds were replaced with the standard control feed. The mortality of fruit flies was observed on the 2nd, 4th, and 8th days post-treatment.

#### 4.2.2. Toxicity Assay to *O. laevigatus*

Spinosad and newly synthesized compounds were tested in a toxicity assay on adults of *O. laevigatus* (Thripor-L, Koppert, Berkel en Rodenrijs, The Netherlands). Petri dishes of 5 cm diameter were sprayed with 150 µL of the tested solutions that were prepared as previously described by Studebaker and Kring [46]. The concentration of all compounds corresponded with the manufacturer’s recommendation for Laser^TM^, which was 120 mg/L, yielding a concentration of 0.09 kg a.i./ha, as previously described [46]. Water and DMSO mix that corresponded with the aforementioned solvent ratio in the stock solution of tested compounds was used as a negative control. Spraying was conducted in a modified Potter tower with the nozzle pressure set at 55 kPa. After drying, the insects were placed in dishes and covered. The Petri dishes were perforated with two air holes secured with gauze. The test was performed in triplicate for every compound, with 5 adult insects per repetition. Mortality was observed 24, 48, and 72 h post-treatment.

### 4.3. Statistical Analysis

The insect mortality from both bioassays was calculated as a percentage:

Mortality (%) = *N* (dead flies)/*N* (flies in treatment) × 100 (2)

Corrected mortality was calculated using Abbott’s formula:

Corrected mortality (%) = [(mortality of treatment (%) − mortality of negative control (%)/(1 − mortality of negative control (%)] × 100 (3)

Data are presented as the mean ± SE of three replicates. One-way analysis of variance was used to compare mean values between the mortality of compounds and the positive control. Fisher’s test was used to determine the statistically significant differences (*p* < 0.05) with Statistica Cloud Software Group, Inc. (Fort Lauderdale, FL, USA) (2023) Data Science Workbench, version 14. TIBCO, San Ramon, CA, USA.

### 4.4. Computational Methods

#### 4.4.1. Ecotoxicological and Environmental Property Calculation

Ecotoxicological and environmental properties were calculated with online software VEGA QSAR (https://www.vegahub.eu/portfolio-item/vega-qsar/) (Available 1 March 2025) [27].

#### 4.4.2. QSAR Method

The three-dimensional structures were optimized using molecular mechanic force fields (MM+) [47], and reoptimized by the semi-empirical AM1 method [48]. The 2D and 3D molecular descriptors used in this study were calculated using ADMEWORKS ModelBuilder 7.9.1.0 (Fujitsu Kyushu Systems Limited, Fukuoka, Japan). Molecular descriptors were calculated with QSARINS-Chem 2.2.1 (University of Insubria, Varese, Italy) [49]. The models were assessed by fitting criteria, internal cross-validation using the leave-one-out (LOO) method, and external validation. The robustness of QSAR models was tested by the Y-randomization test.

For building neural network models, the three molecular descriptors involved in the MLR model were used as input variables. Compounds for the training set (80%) and test set (20%) were selected by the random method. The network type comprised multilayer perceptrons (MLPs), the training algorithm was Broyden–Fletcher–Goldfarb–Shanno (BFGS), and the sum-square error function was used for training neural networks. The networks consist of a minimum of three and a maximum of ten hidden units. The neuron hidden activation function was exponential, and the output activation was tanh.

#### 4.4.3. Molecular Docking

The crystal structures of the receptors and co-crystalized ligand were uploaded from the RCSB Protein Data Bank (https://www.rcsb.org/, accessed on 2 February 2025). The molecular docking was performed with iGEMDOCK (BioXGEM, Hsinchu, Taiwan) using the generic evolutionary method (GA). The GA parameters were set as follows: population size 200; generations 70; number of poses 2; binding site radius 8 Å. The empirical scoring function of iGEMDOCK is (binding energy (kcal/mol) estimated as:

Binding energy = vdW + Hbond + Elec (4)

where the vdW term is van der Waals energy, and Hbond and Elect terms are hydrogen bonding energy and electrostatic energy, respectively.

## 5. Conclusions

In conclusion, our findings provide valuable insights into the insecticidal potential of the chemical compound **2f**. The marked differences in mortality rates between species and exposure methods underscore the complexity of designing effective and environmentally responsible pest management solutions. The pursuit of compounds that exhibit this selectivity is essential for sustainable agriculture. Future research should focus on optimizing the application methods and concentrations of these promising compounds to maximize their efficacy against target pests while minimizing their effects on non-target species, without compromising the health of the ecosystem.

## Figures and Tables

**Table 1:** The results of the toxicity bioassay of coumarin-1,2,4-triazole hybrid compounds on *Drosophila melanogaster* and *Orius laevigatus* (Fieber), presented as a mean value of corrected mortality, % ± standard deviation (SD) of three replications. Mean values and SD were determined via Fisher’s test.

Comp.	*Drosophila melanogaster*			*Orius laevigatus* (Fieber)		
	2 Days	4 Days	8 Days	24 h	48 h	72 h
**1a**	5.28 ± 0.33 *	8.84 ± 2.35 *	27.06 ± 5.56 *			
**1b**	14.42 ± 2.33 *	55.15 ± 10.66 *	95.01 ± 0.85	78.52 ± 1.28	96.30 ± 6.41	100.00 ± 0.00
**1c**	12.56 ± 3.81 *	52.54 ± 14.13 *	84.91 ± 10.53	100.00 ± 0.00	100.00 ± 0.00	100.00 ± 0.00
**1d**	8.78 ± 10.56 *	28.26 ± 13.53 *	47.22 ± 14.97 *			
**1e**	7.71 ± 10.59 *	28.28 ± 6.04 *	63.26 ± 16.54 *			
**1f**	6.60 ± 7.74 *	18.28 ± 9.76 *	39.46 ± 1.81 *			
**1g**	26.67 ± 21.11 *	67.01 ± 21.86 *	92.64 ± 7.08	43.33 ± 5.77 *	64.45 ± 3.85 *	78.57 ± 6.19 *
**1h**	12.82 ± 10.78 *	50.88 ± 9.73 *	70.58 ± 12.30 *			
**1i**	4.72 ± 3.60 *	51.68 ± 17.76 *	86.77 ± 11.46	77.78 ± 19.24	83.33 ± 14.43 *	86.67 ± 11.55 *
**1j**	8.38 ± 8.33 *	26.23 ± 19.32 *	56.86 ± 12.96 *			
**1k**	14.98 ± 7.56 *	60.70 ± 15.24 *	85.58 ± 14.62	70.37 ± 6.41 *	87.96 ± 0.80	92.59 ± 6.41
**2a**	4.52 ± 0.46 *	16.54 ± 7.06 *	28.46 ± 12.62 *			
**2b**	7.07 ± 5.99 *	66.77 ± 27.60 *	89.00 ± 10.10	78.52 ± 1.28	95.83 ± 7.22	100.00 ± 0.00
**2c**	45.40 ± 28.75 *	53.90 ± 31.87 *	56.68 ± 30.02 *			
**2d**	9.94 ± 5.30 *	39.83 ± 8.03 *	59.76 ± 15.24 *			
**2e**	8.08 ± 6.41 *	32.87 ±4.35 *	68.07 ± 1.88 *			
**2f**	60.30 ± 15.35 *	86.36 ± 13.53	100.00 ± 0.00	53.33 ± 28.37 *	61.67 ± 25.04 *	78.57 ± 6.19 *
**2g**	25.06 ± 9.08 *	29.61 ± 11.86 *	26.21 ± 12.51 *			
**2h**	11.07 ± 13.53 *	27.55 ± 6.00 *	54.45 ± 12.47 *			
**2i**	6.62 ± 6.05 *	23.57 ± 7.69 *	41.77 ± 6.87 *			
**2j**	15.39 ± 7.79 *	19.35 ± 2.32 *	20.97 ± 7.28 *			
**2k**	19.84 ± 9.15 *	35.94 ± 6.44	44.87 ± 5.23 *			
**2l**	5.72 ± 6.32 *	16.37 ± 5.51 *	26.68 ± 4.27 *			
**2m**	3.11 ± 4.11 *	10.99 ± 4.91 *	36.84 ± 7.22 *			
**2n**	10.50 ± 4.61 *	71.44 ± 6.80 *	96.02 ± 3.46	96.30 ± 6.42	100.00 ± 0.00	100.00 ± 0.00
**2o**	5.51 ± 2.03 *	15.67 ± 4.90 *	36.05 ± 10.39 *			
**3a**	11.40 ± 9.06 *	30.97 ± 17.43 *	56.89 ± 8.76 *			
**3b**	7.07 ± 5.99 *	20.54 ± 6.23 *	41.08 ± 2.17 *			
**3c**	2.94 ± 2.56 *	8.92 ± 4.50 *	28.08 ± 1.58 *			
**3d**	6.99 ± 8.75 *	17.08 ± 14.17 *	31.54 ± 17.43 *			
**3e**	4.28 ± 0.10 *	5.92 ± 2.75 *	26.34 ± 9.32 *			
**3f**	6.61 ± 3.16 *	8.37 ± 7.63 *	15.15 ± 11.35 *			
**3g**	4.99 ± 4.32 *	19.06 ± 6.09 *	40.26 ± 7.98 *			
spinosad	97.87 ± 3.69	100.00 ± 0.00	100.00 ± 0.00	92.59 ± 12.83	100.00 ± 0.00	100.00 ± 0.00

* Compound results significantly different from positive control (insecticide spinosad) results at *p* < 0.05.

**Table 2:** Predicted environmental fate properties and ecotoxic effects of coumarin-1,2,4-triazole hybrid.

Comp.	BCF ^1^	Fish Acute Toxicity ^2^	Fathead Minnow 96 h ^3^	Zebrafish Embryo ^4^	*Daphnia magna* 48 h ^5^	Algae Acute Toxicity ^6^	Bee Acute Toxicity ^7^	Sludge Toxicity ^8^
**1a**	0.86	4.6	5.9	0.78	6.1	Toxic	Low	Toxic
**1b**	0.75	1.87	4.94	1.91	4.11	Non-toxic	Low	Toxic
**1c**	1.13	4.49	5.46	0.93	4.65	Toxic	Low	Toxic
**1d**	0.93	3.61	5.51	1.2	4.49	Non-toxic	Low	Toxic
**1e**	1.22	2.83	5.92	1.1	4.58	Toxic	Low	Non-toxic
**1f**	1.21	2.83	5.91	1.11	4.63	Toxic	Low	Non-toxic
**1g**	1.26	2.83	5.98	1.01	4.75	Toxic	Low	Toxic
**1h**	0.99	2.29	5.52	1.21	4.58	Non-toxic	Low	Toxic
**1i**	1.19	4.49	5.79	1.02	4.56	Toxic	Low	Non-toxic
**1j**	0.66	1.87	4.62	2.18	3.98	Non-toxic	Low	Toxic
**1k**	1.09	2.83	5.48	1.03	4.46	Toxic	Low	Non-toxic
**2a**	0.56	1.87	4.81	0.78	5.51	Toxic	Low	Toxic
**2b**	0.72	2.83	5.33	0.34	5.72	Toxic	Low	Toxic
**2c**	1.08	2.29	5.81	0.65	6.27	Toxic	Low	Toxic
**2d**	0.86	4.6	5.9	0.78	6.1	Toxic	Low	Toxic
**2e**	1.28	2.29	6.31	0.82	6.26	Toxic	Low	Toxic
**2f**	1.31	2.29	6.36	0.73	6.99	Toxic	Low	Toxic
**2g**	1.35	2.29	5.99	0.97	6.22	Toxic	Low	Toxic
**2h**	1.3	4.8	6.49	−0.21	6.52	Toxic	Low	Toxic
**2i**	0.91	4.6	5.87	0.62	6.18	Toxic	Low	Toxic
**2j**	1.07	2.29	6.14	0.33	6.22	Toxic	Low	Toxic
**2k**	0.62	2.49	5.02	0.6	5.58	Toxic	Low	Toxic
**2l**	1.02	4.49	5.84	0.44	6.11	Toxic	Low	Toxic
**2m**	1.29	2.29	6.33	0.57	7.08	Toxic	Low	Toxic
**2n**	1.28	2.29	6.27	0.66	6.31	Toxic	Low	Toxic
**2o**	0.42	1.87	4.67	0.64	5.51	Toxic	Low	Toxic
**3a**	1.03	2.29	5.48	−0.36	6.24	Toxic	Low	Toxic
**3b**	0.86	4.6	5.54	−0.39	6.13	Non-toxic	Low	Toxic
**3c**	1.07	2.29	5.94	−0.35	6.29	Toxic	Low	Toxic
**3d**	1.31	2.29	5.66	−0.04	6.19	Toxic	Low	Non-toxic
**3e**	1.27	4.8	6.16	0.4	6.46	Toxic	Low	Non-toxic
**3f**	1.28	4.49	6.03	−0.27	7.02	Toxic	Low	Non-toxic
**3g**	0.33	1.87	4.34	1.52	5.55	Toxic	Strong	Non-toxic

^1^ Bioconcentration [log(L/kg)]; ^2^ pLC_50_ NIC model, the concentration required to kill 50% of fish within 96 h [log(1/(mmol/L))]; ^3^ pLC_50_ 96 h EPA model (mol/L); ^4^ developmental toxicity towards zebrafish embryo, half-maximal activity concentration (AC_50_) [log(µmol/L)]; ^5^ pLC_50_ 48 h EPA model (mol/L); ^6^ ProtoQSAR-Combase prediction, binary classification: toxic (ErC_50_ < 10 mg/L), Non-toxic (ErC_50_ ≥ 10 mg/L); ^7^ kNN/IRFMN model 48 h LD_50_ (μg/bee); low toxicity (>100 µg/bee); strong toxicity (<1 μg/bee); ^8^ The effects of a substance on microorganisms from activated sludge.

**Table 3:** The summary of the ANN model.

Network	*R* ^2^_training_	*R* ^2^_test_	Training Error	Test Error
MLP 3-4-1	0.78	0.94	0.02	0.02

**Table 4:** Global sensitivity of variables included in the MLP-ANN model 3-4-1.

Network	*nRCt(sp2)*	*PW2*	*RDF075m*
MLP 3-4-1	13.99	1.47	1.35

**Table 5:** The five best docking score energies (kcal/mol) of coumarin-1,2,4-triazole hybrids on the GluClcryst–Fab complex, (pdb: 3RIF), *D. melanogaster* dopamine transporter (dDAT) (pdb: 7WGT), and acetylcholine binding protein from *Aplysia californica* (Ac-AChBP) (pdb: 4ZK4), along with the co-crystallized ligands (ivermectin, **IVM**; glutamate, **Glu; NO-711**; **TII**), and an active component of insecticide (spinosyn A, **SPYNA**).

Receptor	GluCl				dDAT		Ac-AChBP	
Binding Site	IVM		Glu		NO-711		TII	
	Comp.	Total E	Comp.	Total E	Comp.	Total E	Comp.	Total E
	**IVM**	−142.87	**2c**	−113.45	**NO-711**	−114.61	**TII**	−116.98
	**2d**	−104.82	**2i**	−109.52	**1h**	−113.96	**3a**	−103.63
	**2f**	−103.21	**2n**	−107.68	**1g**	−108.60	**2c**	−102.31
	**2e**	−103.16	**2b**	−107.56	**2h**	−101.39	**1a**	−100.51
	**3a**	−99.38	**2j**	−106.94	**1c**	−98.97	**2a**	−97.81
	**3c**	−99.38	**2m**	−106.31	**2c**	−97.80	**2b**	−97.62
	**SPYNA**	−79.89	**2o**	−105.96	**SPYNA**	−44.16	**SPYNA**	−50.70

## Data Availability

The authors confirm that the data supporting the findings of this study are available within the article.

## Abbreviations

The following abbreviations are used in this manuscript:

QSAR	Quantitative structure–activity relationship
AChE	Acetylcholinesterase
nAChR	Nicotinic acetylcholine receptor
GABA	γ-aminobutyric acid
GluCl	Glutamate-gated chloride channel
MLR	Multiple linear regression
ANN	Artificial neural network
dDAT	*Drosophila melanogaster* dopamine transporter
MLP	Multilayer perceptron
